# An anti-CD30 single-chain Fv selected by phage display and fused to *Pseudomonas* exotoxin A (Ki-4(scFv)-ETÁ) is a potent immunotoxin against a Hodgkin-derived cell line

**DOI:** 10.1038/sj.bjc.6690488

**Published:** 1999-06

**Authors:** A Klimka, S Barth, B Matthey, R C Roovers, H Lemke, H Hansen, J-W Arends, V Diehl, H R Hoogenboom, A Engert

**Affiliations:** Laboratory of Immunotherapy, Department of Internal Medicine I, Joseph Stelzmann Str. 9, Cologne, 50931, Germany; Department of Pathology, CESAME, PO Box 5800, Maastricht, AZ, 6202, The Netherlands; , Biochemical Institute of the Christian Albrechts University Kiel, Olshausenstr. 40, Kiel, 24118, Germany

**Keywords:** CD30, Ki-4, phage display, recombinant immunotoxin, Hodgkin's lymphoma

## Abstract

The human CD30 receptor is highly overexpressed on the surface of Hodgkin Reed-Sternberg cells and has been shown to be an excellent target for selective immunotherapy using monoclonal antibody-based agents such as immunotoxins. To construct a new recombinant immunotoxin for possible clinical use in patients with Hodgkin's lymphoma, we have chosen the murine anti-CD30 hybridoma Ki-4 to generate a high-affinity Ki-4 single-chain variable fragment (scFv). Hybridoma V-genes were polymerase chain reaction-amplified, assembled, cloned and expressed as a mini-library for display on filamentous phage. Functional Ki-4 scFv were obtained by selection of binding phage on the Hodgkin lymphoma-derived, CD30-expressing cell line L540Cy. The selected recombinant Ki-4 scFv was shown to specifically bind to an overlapping epitope on the CD30 antigen with binding kinetics similar to those of the original antibody. The Ki-4 scFv was subsequently fused to a deletion mutant of *Pseudomonas* exotoxin A (ETÁ). The resulting immunotoxin Ki-4(scFv)-ETÁ specifically binds to CD30^+^ L540Cy cells and inhibits the protein synthesis by 50% at a concentration (IC_50_) of 43 pM. This recombinant immunotoxin is a promising candidate for further clinical evaluation in patients with Hodgkin's lymphoma or other CD30^+^ malignancies. © 1999 Cancer Research Campaign


*British Journal of Cancer* (1999) **80**(8), 1214–1222
© 1999 Cancer Research Campaign
Article no. bjoc.1998.0488

### An anti-CD30 single-chain Fv selected by phage display and fused to *Pseudomonas* exotoxin A (Ki-4(scFv)-ETA´) is a potent immunotoxin against a Hodgkin-derived cell line

**A Klimka[1,2], S Barth[1], B Matthey[1], RC Roovers[2], H Lemke[3], H Hansen[3], J-W Arends[2], V Diehl[1], HR Hoogenboom[2] and A Engert[1]**

[1]Laboratory of Immunotherapy, Department of Internal Medicine I, University Hospital Cologne, Joseph Stelzmann Str. 9, 50931 Cologne, Germany; [2]CESAME, Department of Pathology, University Hospital Maastricht, PO Box 5800, 6202 AZ Maastricht, The Netherlands; [3]Biochemical Institute of the Christian Albrechts University Kiel, Olshausenstr. 40, 24118 Kiel, Germany

**Summary** The human CD30 receptor is highly overexpressed on the surface of Hodgkin Reed-Sternberg cells and has been shown to be an excellent target for selective immunotherapy using monoclonal antibody-based agents such as immunotoxins. To construct a new recombinant immunotoxin for possible clinical use in patients with Hodgkin's lymphoma, we have chosen the murine anti-CD30 hybridoma Ki-4 to generate a high-affinity Ki-4 single-chain variable fragment (scFv). Hybridoma V-genes were polymerase chain reaction-amplified, assembled, cloned and expressed as a mini-library for display on filamentous phage. Functional Ki-4 scFv were obtained by selection of binding phage on the Hodgkin lymphoma-derived, CD30-expressing cell line L540Cy. The selected recombinant Ki-4 scFv was shown to specifically bind to an overlapping epitope on the CD30 antigen with binding kinetics similar to those of the original antibody. The Ki-4 scFv was subsequently fused to a deletion mutant of *Pseudomonas* exotoxin A (ETA´). The resulting immunotoxin Ki-4(scFv)-ETA´ specifically binds to CD30$^+$ L540Cy cells and inhibits the protein synthesis by 50% at a concentration ($IC_{50}$) of 43 pM. This recombinant immunotoxin is a promising candidate for further clinical evaluation in patients with Hodgkin's lymphoma or other CD30$^+$ malignancies.

**Keywords:** CD30; Ki-4; phage display; recombinant immunotoxin; Hodgkin's lymphoma

Hodgkin's lymphoma is one of the best suited malignancies for targeted immunotherapy for the following reasons: (1) Hodgkin Reed-Sternberg (H–RS) cells have surface markers such as CD25 and CD30, which are present only on a minority of normal lymphoid cells; (2) The number of malignant cells that needs to be killed is small since the majority of cells in Hodgkin's lymphoma are non-malignant reactive cells; (3) Hodgkin tumours are usually well-vascularized, suggesting that access of an immunotherapeutic agent like an immunotoxin (IT) to the target cells should be easier than in solid tumours; (4) Although Hodgkin's lymphoma is known to respond well to chemotherapy, residual tumour cells remaining after first-line treatment have been demonstrated to correlate with the probability of a later relapse. As the selective elimination of residual H–RS cells might enhance the number of patients being cured, it seems feasible to eradicate bulky disease by conventional therapy first and then to administer immunotoxins to kill residual H–RS cells.

One problem associated with the use of immunotoxins is the heterogeneity of antigen expression on tumour cells and therefore the selection of antigen-negative tumour cells (Engert et al, 1990; Vitetta et al, 1993). This problem can be circumvented by the application of immunotoxin cocktails, i.e. mixtures of immunotoxins against different antigens on the same target cell. Immunotoxin cocktails have demonstrated better effects as compared to the use of single immunotoxins in animal lymphoma models (Ghetie et al, 1992). In addition, a cocktail consisting of immunotoxins against different antigens has given superior results against Hodgkin's lymphoma, both in vitro and in vivo (Engert et al, 1995). Thus, our group has developed a variety of immunotoxins for possible clinical use in Hodgkin's lymphoma, like anti-CD25, anti-CD30 and anti-interleukin (IL)-9 receptor immunotoxins (Engert et al, 1990, 1997; Klimka et al, 1996).

One of the most promising candidate target antigens for immunotherapeutics against Hodgkin's disease (HD) is CD30. The CD30 antigen was originally discovered on cultured H–RS cells using the monoclonal antibody (mAb) Ki-1 (Schwab et al, 1982). Expression screening permitted the cloning of the gene that encodes for the CD30 receptor molecule (Dürkop et al, 1992), which is located on chromosome 1p36. Subsequently, the naturally occurring CD30 ligand was identified and cloned (Gruss et al, 1995). The CD30/CD30 ligand system can trigger cytolytic cell death in malignant lymphoma cell lines and has been demonstrated to induce proliferation and cytokine production in T-cells or neutrophils (Wiley et al, 1996). Since CD30 is present in high copy numbers on malignant lymphoma cells and has a very limited distribution on normal cells, mAbs against CD30 have been explored as vehicles for cytostatic drugs (Sahin et al, 1990) or plant toxins (Terenzi et al, 1996). Immunotoxins constructed with anti-CD30 mAbs have demonstrated specificity and potent anti-tumour activity against Hodgkin's lymphoma cells in vitro and in mouse models (Engert et al, 1990; Schnell et al, 1995). A clinical trial has been reported using an immunotoxin constructed by

*Received 3 September 1998*
*Revised 27 November 1998*
*Accepted 7 December 1998*

*Correspondence to:* A Engert

A Klimka and S Barth contributed equally to the work.

chemically linking the anti-CD30 mAb BerH2 to Saporin-6 (Pasqualucci et al, 1995). Twelve patients with advanced refractory Hodgkin's lymphoma were enrolled and five patients exhibited a rapid reduction in tumour masses, underlining the validity of CD30 as a target antigen in HD.

The problems identified in these and other clinical trials with immunotoxins are the development of antibodies against the immunotoxins neutralizing their effects (Grossbard et al, 1993; Vitetta et al, 1993), liver toxicity and vascular leak syndrome. In addition, there are difficulties in producing large quantities of chemically linked immunotoxins for randomized clinical trials. These problems might, at least in part, be overcome by using recombinant DNA technology which makes the construction of less immunogenic and smaller immunotoxins feasible, and more easily permits the production of immunotoxins in large quantities. It is also believed that penetration into tumours should be better for small proteins than large conjugates. Recombinant immunotoxins can be engineered by fusing only the small antigen-binding part of a whole antibody to bacterial or plant toxin genes, lacking their binding domains (reviewed in Kreitman and Pastan, 1994).

In this paper, we report the successful cloning of the variable genes of the recently evaluated anti-CD30 mAb Ki-4, which has been shown to inhibit the shedding of the extracellular part of the CD30 molecule (Horn-Lohrens et al, 1995). A chemically linked ricin A-chain immunotoxin (Ki-4-SMPT-dgA) constructed with Ki-4 mAb exhibited powerful specific cytotoxicity ($IC_{50}$ of 40 pM) against $CD30^+$ tumour cells in vitro and cured 60% of severe combined immune deficient (SCID) mice treated with a single injection of 8 μg (Schnell et al, 1995). The phage display technology made it possible to get access to a specific, high-affinity Ki-4 single-chain variable fragment (scFv) which was further genetically fused to a modified *Pseudomonas* exotoxin A gene (ETA´). We demonstrate that the new recombinant immunotoxin constructed with the selected scFv, Ki-4(scFv)-ETA´, is specific and highly effective in destroying a $CD30^+$ Hodgkin-derived cell line. This scFv-based immunotoxin is a good candidate on its own, or in combination with other immunotoxins, for immunotherapy of Hodgkin's disease.

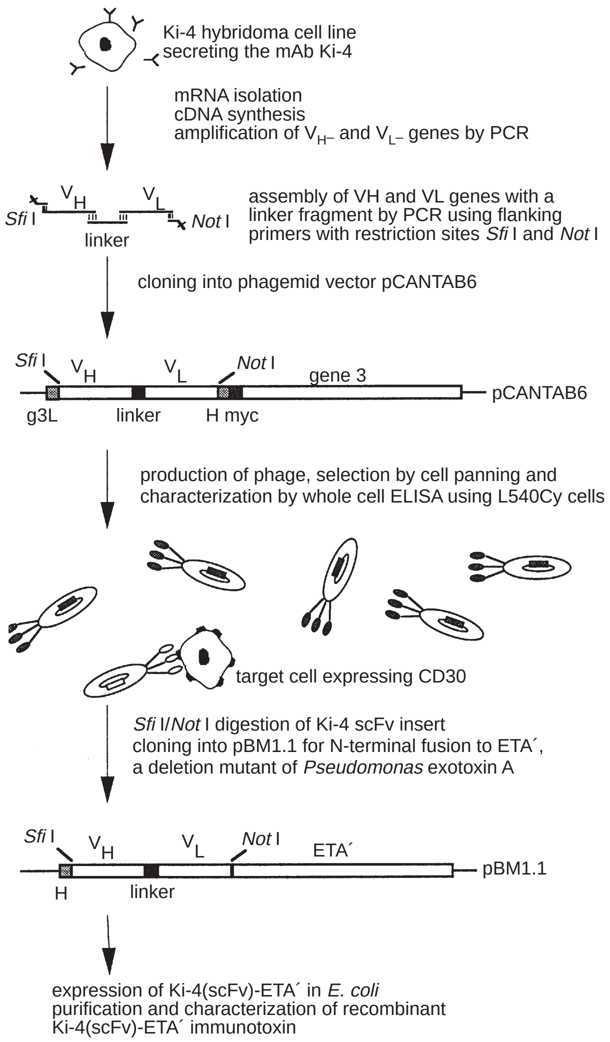

**Figure 1** Cloning schedule of Ki-4(scFv)-ETA´. Phagemid vector pCANTAB6 contains a $His_6$-tag (H) for immobilized metal ion chromatography (IMAC) purification of the scFv and a c-myc-tag (myc) for its detection using an anti-myc antibody. The g3L sequence encodes for a signal peptide guiding the native protein into the periplasm and gene 3 encodes the phage coat protein p3

### MATERIALS AND METHODS

### Cell lines

The Hodgkin-derived cell line L540Cy, the Burkitt lymphoma cell line BL38, and the hybridoma cell lines Ki-3 and Ki-4 (Horn-Lohrens et al, 1995) were maintained in RPMI-1640 medium (Gibco) supplemented with 10% (v/v) fetal calf serum (FCS), 100 μg $ml^{-1}$ streptomycin, 200 units $ml^{-1}$ penicillin and 2 mM L-glutamine. Transfected baby hamster kidney (BHK)-21 cells secreting a construct of the extracellular domain of the human CD30 receptor fused to the hinge, CH2 and CH3 domain of human IgG1 (sCD30-Fc) were kindly provided by H-J Gruss (Ulm, Germany) and maintained in RPMI-1640 medium supplemented with 10% (v/v) IgG-free FCS (c.c. pro GmbH) and 1 mg $ml^{-1}$ gentamycin (PAA). All cells were cultured at 37°C in a 5% carbon dioxide atmosphere.

### Bacterial strains and plasmids

*Escherichia coli* TG-1 (K12 Δ(*lac-pro*), *supE*, *thi*, *hsd*D5/F´*tra*D36, *pro*$A^+B^+$, *lacI*$^q$, *lac*ZΔM15) and *E. coli* HB2151 (K12 Δ(*lac-pro*), *ara*, *nal*$^r$, *thi*/F´, *proA*$^+B^+$, *lacI*$^q$, *lac*ZΔM15) were purchased from Pharmacia. The phagemid vector pCANTAB6 (McCafferty et al, 1994) is derived from pHEN1 (Hoogenboom et al, 1991), carrying an additional $His_6$-tag for immobilized metal-ion affinity chromatography (IMAC) purification and a myc-tag for detection by anti-myc-tag antibody 9E10 (Munro et al, 1986). It is used for N-terminal fusion of *Sfi*I/*Not*I-scFv fragments to the minor coat protein p3 of filamentous phage M13 (Figure 1). The plasmid pBM1.1 (Matthey et al, 1999) is derived from the pET27b vector (Novagen) and is used for N-terminal fusion of scFvs to a modified deletion mutant of *Pseudomonas aeruginosa* exotoxin A (Wels et al, 1992), detectable by anti-ETA mAb TC-1 (kindly provided by DR Galloway, Ohio, USA).

### Cloning of the murine Ki-4 scFv

Total cellular RNA from $10^7$ cells of the Ki-4 hybridoma cell line was isolated using the RNazol™ solution (Biotecx) as described

**Figure 2** Sequence of Ki-4 scFv with deduced amino acids. Nucleotides derived from primers are underlined. The complementarity determining regions (CDR) and the $(Gly_4Ser)_3$ linker between the heavy and light chain are indicated by the manufacturer. cDNA was synthesized using 5 µg of freshly prepared RNA and 10 µl random hexamer primers (10 µM) of the RiboClone cDNA Synthesis Systems kit (Promega) in a 50 µl reaction mix. VH and VL genes were amplified from 5 µl of cDNA using 20 pmol of each primer VH1BACK/VH1FOR-2 and VKBACKmix/VKFOR4mix (Roovers et al, 1998) and assembled with a linker sequence $(Gly_4Ser)_3$ according to Marks et al (1991) (Figure 1). Assembly products were gel-purified and phenol-extracted. Purified fragments were cloned into the phagemid vector pCANTAB6 using the restriction sites *Sfi*I/*Not*I. The ligation mix was purified by phenol extraction, precipitated with ethanol, and dissolved in 10 µl water. The DNA solution was transfected into 50 µl *E. coli* TG-1 by electroporation as described elsewhere (Dower et al, 1988). The cells were grown for 1 h in 2 × TY medium at 37°C before plating on 2 × TY agar medium containing 100 µg ampicillin $ml^{-1}$ and 2% (w/v) glucose (2 × TY-Amp-Glu).

### Selection of phage particles on the Hodgkin cell line L540Cy

A repertoire of transformed bacteria (approximately $10^5$ clones) containing scFv polymerase chain reaction (PCR) products ligated into pCANTAB6 was rescued with helper phage M13K07 as described (Marks et al, 1991). Five times $10^5$ L540Cy cells were incubated with 1 ml of $1 \times 10^{13}$ colony forming units (cfu) per ml phage in 2% (w/v) MPBS (2% Marvel – skimmed milk powder – in PBS) for 1 h at room temperature (RT) on a rotating turntable. After washing the cells ten times with 5 ml 2% MPBS and two times with 5 ml PBS by spinning (300 ***g***, 3 min, RT) and resuspending respectively, binding phage were eluted using 1.5 ml 50 mM hydrochloric acid for 10 min on a turntable. Eluted material was immediately neutralized by adding 0.5 ml 1 M Tris–HCl (pH 7.5) and remaining cell debris was spun down (300 ***g***, 5 min, RT). Phage-containing supernatant was mixed with 3 ml 2 × TY-Glu medium and used to transfect 5 ml logarithmically growing *E. coli* TG-1 cells for 30 min at 37°C before plating them on 2 × TY-Amp-Glu agar medium.

### Whole cell ELISA

A total of $1 \times 10^7$ L540Cy cells were resuspended in 10 ml PBS containing 2% (w/v) bovine serum albumin (BSA) (PBSA 2%). Then, 100 µl of cellular suspension were filled in each well of a V-bottom microtitre plate (Greiner) and centrifuged (500 ***g***, 3 min, RT). Supernatant was removed by flicking the plate. Cell pellets were resuspended in 150 µl PBSA 2% and incubated for 1 h at RT. Cells were centrifuged (500 ***g***, 3 min, RT), supernatant was discarded, and cells were resuspended in 150 µl PBS containing 0.1% (w/v) BSA (PBSA 0.1%). This washing step was repeated

 *© 1999 Cancer Research Campaign*

once and cells were resuspended in 50 μl of soluble fragment-containing supernatants of induced bacterial clones according to Marks et al (1991). After 1 h incubation the L540Cy cells were washed three times in 150 μl PBSA 0.1% and subsequently incubated for 1 h in 50 μl of one-fifth-diluted hybridoma supernatant of the anti-myc-tag antibody 9E10. Cells were washed three times in 150 μl PBSA 0.1% and resuspended in 50 μl of a 1/5000-diluted rat anti-mouse IgG peroxidase-conjugated antibody (Dianova). After 30 min incubation at RT, cells were washed four times in 150 μl PBS, transferred to a new V-bottom 96-well plate and incubated with 50 μl peroxidase-substrate. After incubation for approximately 15 min, the reaction was stopped by addition of 50 μl $H_2SO_4$ (2 N) and cells were centrifuged again. Extinction at 490 nm was measured with an enzyme-linked immunosorbent assay (ELISA) reader (MWG) after transferring the supernatants to a flat-bottom microtitre plate. Clones were considered positive for CD30-binding if the $OD_{490}$ was at least three times higher than the background.

### Cell-binding activity of Ki-4 scFv

Five times $10^5$ L540Cy cells were washed in 2% (w/v) MPBS containing 0.05% (w/v) sodium azide (2% MPBS/$N_3$–), and then incubated for 1 h at 4°C with approximately $10^{10}$ cfu of phage displaying Ki-4 scFv and unpurified supernatant from hybridomas secreting mAb Ki-3 or mAb Ki-4 respectively. Bound phage were detected with a sheep anti-fd serum (Pharmacia; 0.02% (v/v) in 2% MPBS/$N_3$–) and fluorescein isothiocyanate (FITC)-labelled rabbit-anti-sheep IgG (Dianova; 2% (v/v) in PBS/$N_3$–). Appropriate amounts of stained cells were brought onto slides by cytocentrifugation (300 ***g***, 10 min), fixed using a 1/9-DABCO-(20% 1,4 diazabicyclo[2.2.2.]octane (Sigma) in 0.2 M Tris–HCl, pH 8, 0.02% $NaN_3$–) DAPI-(0.5 μg 4,6-diamidino-2-phenylindole (Sigma) in 100% glycerol) solution and subsequently analysed on a fluorescence microscope (Leica).

### Purification of Ki-4 scFv

*E. coli* HB2151, harbouring the Ki-4 scFv in pCANTAB6, was used to inoculate 250 ml of 2 × TY-Amp-Glu. The culture was grown at 37°C to an $OD_{600}$ of 0.9, subsequently centrifuged and resuspended in 50 ml 2 × TY-Amp with 1 mM isopropyl-β-*D*-galactopyranoside (IPTG) for induction of soluble scFv expression. After 5 h of induction, the cells were placed on ice for 20 min, pelleted and resuspended in 5 ml ice cold PBS with 1 M sodium chloride and 1 mM EDTA in total. After incubation for 30 min on ice, the suspension was centrifuged (3000 ***g***, 10 min, RT) to remove bacteria and the resulting periplasmic fraction was subsequently centrifuged for 10 min at 25 000 ***g*** to remove cell debris. The scFvs were further purified by IMAC using 2 ml Ni-NTA resin (Qiagen) according to the manufacturer's instructions. Eluted protein was thoroughly dialysed against PBS and checked on sodium dodecyl sulphate polyacrylamide gel electrophoresis (SDS-PAGE) and by immunoblotting. The final concentration was determined from a scanned Coomassie-stained SDS-PAGE using the Molecular Analyst software (Bio-Rad).

### Kinetic measurement using SPR in a BIAcore

Binding kinetics of the Ki-4 mAb and the Ki-4 scFv were determined performing surface plasmon resonance (SPR) in a BIAcore 2000 (Pharmacia) using recombinant, human sCD30-Fc protein, which was purified from supernatant of stable transfected BHK cells on an anti-human IgG resin (Sigma) according to the manufacturer's instructions. sCD30-Fc protein was covalently coupled to a CM-5 sensorchip (Pharmacia) via free amide chemistry, resulting in a surface of 450 resonance units (RU). This 'low density' CD30 surface was saturated with antibody or antibody fragment using multiple injections of protein and a flow rate of 20 μl $min^{-1}$. Dissociation rates were then determined by curve fitting from the resulting sensorgrams using the BIAevaluation software (Pharmacia).

A

B

C

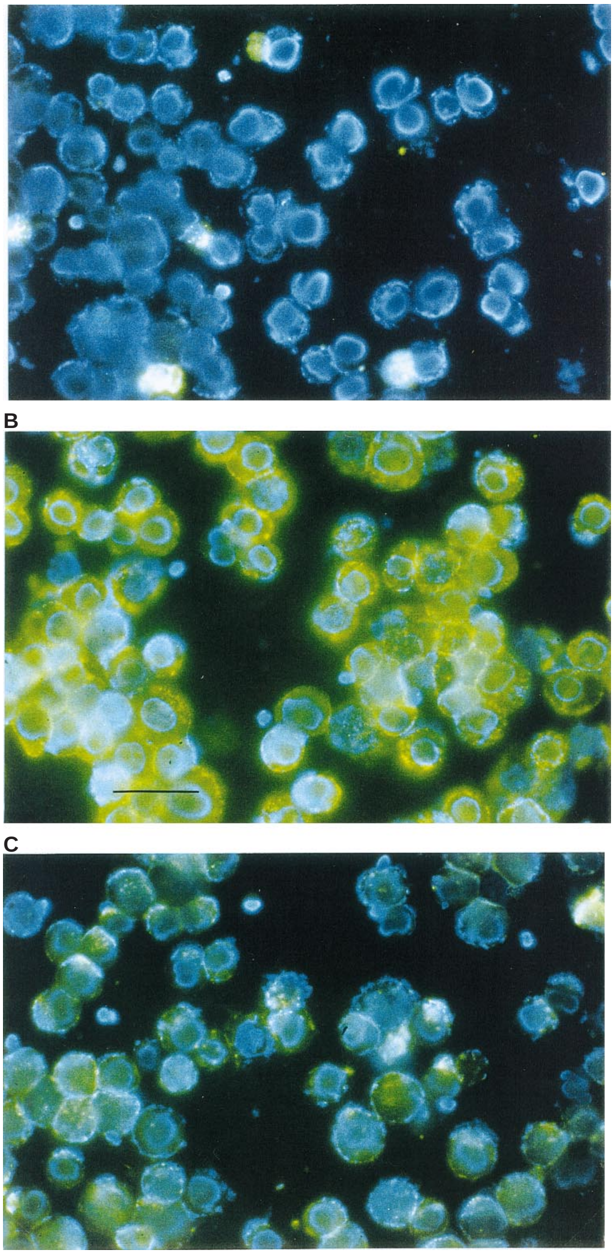

**Figure 3** Competitive immunofluorescence staining of L540Cy cells with Ki-4 scFv displayed on phage. Cells were incubated with (**A**) phage displaying no recombinant protein, (**B**) phage displaying Ki-4 scFv on their surface and supernatant of Ki-3 hybridoma for competition, (**C**) phage displaying Ki-4 scFv and supernatant of Ki-4 hybridoma for competition. Binding of phage was detected with sheep anti-fd serum and FITC-labelled rabbit-anti-sheep IgG. Cells were centrifuged on slides and fixed with DABCO/DAPI in glycerol, giving a blue nuclear staining. Scale bar: 45 μm

© 1999 Cancer Research Campaign

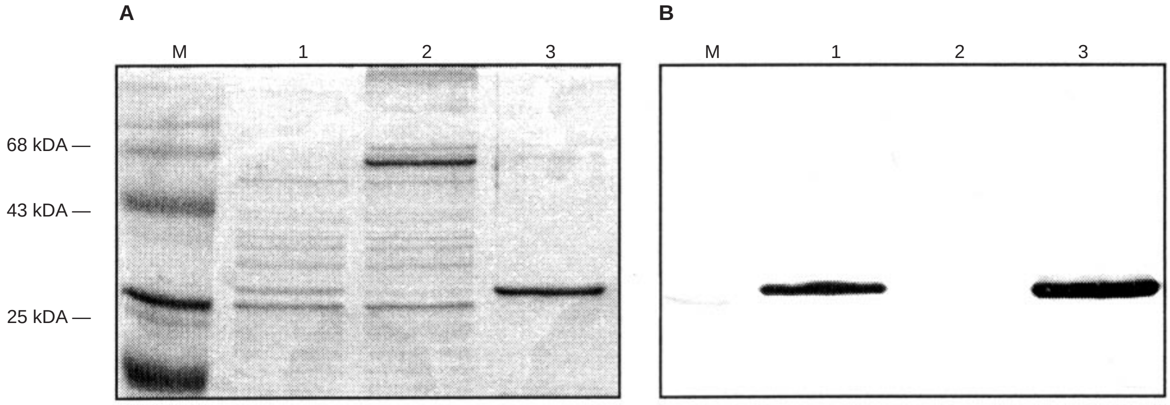

**Figure 4** Purification of Ki-4 scFv by IMAC. SDS-PAGE gel was stained with Coomassie (**A**) and a Western blot (**B**) was performed, using a mouse anti-myc-tag mAb, and anti-mouse mAb conjugated with streptavidin-alkaline phosphatase, visualized by phosphatase substrate. Lane M, prestained molecular weight marker; lane 1, periplasmic extraction; lane 2; flow through of Ni-NTA resin; lane 3, eluate of Ni-NTA resin

### Construction and purification of the recombinant anti-CD30 immunotoxin Ki-4(scFv)-ETA´

Ki-4 (scFv) gene was released from phagemid vector pCANTAB6 by *Sfi*I/*Not*I digestion and inserted into *Sfi*I/*Not*I digested expression vector pBM1.1 containing a modified *Pseudomonas* exotoxin A (ETA´)-gene (Wels et al, 1992). The resulting pBM1.1-Ki-4(scFv)-ETA´ plasmid was transformed into *E. coli* BL21 (DE3) (Novagen). Recombinant Ki-4(scFv)-ETA´ was purified from inclusion bodies and refolded with the help of 6 M guanidinium hydrochloride according to a previously described protocol (Plaksin et al, 1996). Purification was performed by ion-exchange chromatography using a mono-Q column (Pharmacia). Protein concentration was determined by SDS-PAGE and Coomassie staining, using BSA standards and densitometrical analysis with Molecular Analyst software (Bio-Rad).

### Binding activity and cytotoxicity of Ki-4(scFv)-ETA´

Binding specificity of Ki-4(scFv)-ETA´ was demonstrated by means of FACS analysis using a FACScan (Becton Dickinson). Target cells were washed in 2% (w/v) MPBS containing 0.05% (w/v) sodium azide (2% MPBS/$N_3$) and incubated with 50 µl of Ki-4(scFv)-ETA´ (10 µg $ml^{-1}$) in 2% MPBS/$N_3$ for 1 h at 4°C. Bound Ki-4(scFv)-ETA´ was detected with the anti-ETA mAb TC-1 (20% (v/v) hybridoma supernatant in 2% MPBS/$N_3$) and FITC-conjugated goat-anti-mouse IgG (Becton Dickinson; 2% (v/v) in PBS/$N_3$).

The effect of Ki-4(scFv)-ETA´ on the protein synthesis of target cell lines was determined by measurement of [$^3$H]-leucine incorporation as described (Barth et al, 1998). A competitive protein synthesis inhibition assay was performed by adding 10 µg $ml^{-1}$ purified Ki-4 mAb or 10 µg $ml^{-1}$ OKT3 moab (Cilac) as a control to a dilution series of Ki-4(scFv)-ETA´.

### RESULTS

### Cloning of V-genes and selection of the Ki-4 scFv

After extraction of total RNA from the hybridoma cell line Ki-4, the variable domains of heavy (VH) and light (VL) chain immunoglobulin genes were amplified by means of reverse transcription (RT)-PCR using an improved set of oligonucleotides (Roovers et al, 1998) and assembled by Splice Overlap Extension (SOE-) PCR (Marks et al, 1991) with a $(Gly_4Ser)_3$ linker. The resulting single-chain variable fragment (scFv) was cloned into phagmid vector pCANTAB6 for expression as a fusion product with the bacteriophage coat protein p3 (Figure 1) or as a soluble scFv fragment, depending on the expression conditions (Marks et al, 1991). The first screening of soluble scFv fragments produced by 94 different bacterial clones in a whole cell ELISA using the CD30-positive Hodgkin-derived cell line L540Cy revealed no binders. To retrieve a functional scFv from the hybridoma cell line Ki-4, phage particles were prepared from approximately $10^5$ bacterial clones containing scFv cloning products. Two successive rounds of selection on L540Cy cells were performed to enrich the population to finally 95% L540Cy binding scFv clones determined in a whole cell ELISA (data not shown).

### V gene sequences of Ki-4 scFv

The DNA sequences of VH and VL chain from two selected murine Ki-4 scFv were determined using a semi-automated ALFexpress sequencer (Pharmacia) and fluorescence-labelled primers, annealing downstream of the scFv cassette in the gene 3 of pCANTAB6 or upstream of the scFv cassette in the pCANTAB6 backbone. The determined nucleic acid sequence and its deduced amino acid sequence are depicted in Figure 2. The nucleic acid sequences of the V regions were compared to the Kabat database of murine V-genes (http://immuno.bme.nwu.edu/famgroup.html) to determine the V gene family. The VH gene segment belongs to the Kabat VII and the VL gene to the Kabat XXI family. The scFv sequence was submitted to GenBank (accession number AF002242).

### Binding properties of Ki-4 scFv

Binding of Ki-4 scFv to the L540Cy cells was demonstrated by means of immunofluorescence staining (Figure 3). To verify binding specificity of the Ki-4 scFv to the CD30-epitope recognized by the Ki-4 mAb, competition experiments were performed. As shown in Figure 3C, a reduction in green immunofluorescence staining is visible if Ki-4 scFv displayed on phage was simultaneously incubated with the parental Ki-4 mAb. In contrast, when the Ki-3 mAb was used, which recognizes a different, non-overlap-

 *© 1999 Cancer Research Campaign*

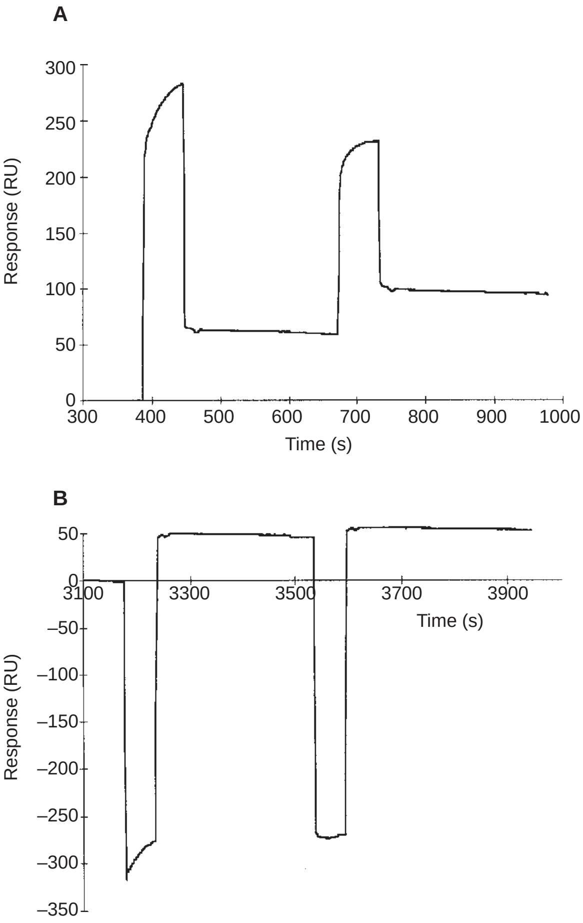

**Figure 5** Kinetic measurement using the BIAcore. Sensorgrams showing surface plasmon resonance (SPR) measurements of Ki-4 mAb (**A**) and Ki-4 scFv (**B**) towards covalently coupled sCD30-Fc protein. Off-rates were determined by curve fitting on the time intervals 800–810 s (**A**) and 3850–3860 s (**B**) respectively

**Table 1** Kinetic data of Ki-4 whole antibody and Ki-4 scFv

| Antibody | $k_{off}$ ($10^{-4}$ $s^{-1}$) ± SE (monovalent) | $k_{off}$ ($10^{-4}$ $s^{-1}$) ± SE (bivalent) | $t^1/_2$ (=ln2/$k_{off}$) |
|---|---|---|---|
| Ki-4 mAb | – | 1.2 ± 0.2 | 96 min |
| Ki-4 scFv | 1.8 ± 0.5 | – | 64 min |

The kinetic data were evaluated by surface plasmon resonance measurements in a BIAcore 2000 using recombinant, human sCD30-Fc protein as antigen. The off-rates were determined from the sensorgrams in Figure 5. $k_{off}$, off-rate; $t^1/_2$, half-life of antibody–antigen complex.

ping epitope on the CD30 antigen, no reduction in fluorescence intensity was observed (Figure 3B). A control experiment with phage displaying no scFv on their surface exhibits only the blue nuclear background staining of the L540Cy cells, due to treatment of the cells with DAPCO/DAPI (Figure 3A). The Ki-4 scFv-gene in pCANTAB6 was subsequently introduced in *E. coli* non-suppressor strain HB2151, which does not translate the amber-stop codon between the scFv- and the p3-gene, and therefore solely expresses soluble scFv. After induction with IPTG, His-tagged Ki-4 scFv produced from HB2151 cells was purified from periplasmic fraction using a Ni-chelate resin. The Ki-4 scFv was recovered in a highly purified form (purity > 90%; Figure 4A) and identified by Western blot-analysis as a 27-kDa band (Figure 4B). The typical yield of purified scFv was approximately 200 μg $l^{-1}$ bacterial culture.

One of the most important characteristics of antibodies to be used in tumour targeting is the rate with which they detach from bound antigen on the cell, determined in part by the off-rate of the molecule (Adams et al, 1993). We compared the off-rates of Ki-4 whole antibody and Ki-4 scFv performing SPR in a BIAcore 2000 (Figure 5 and Table 1) with purified sCD30-Fc protein. As can be seen in Table 1, the off-rates differ by a factor of 1.5 only. The predicted half-life of the antibody–antigen complex was calculated as $t^1/_2$ = ln2/$k_{off}$, resulting in 96 min for the whole antibody and 64 min for the antibody fragment.

### Construction and purification of the recombinant Ki-4(scFv)-ETA´ immunotoxin

After recloning of the Ki-4 scFv gene into the expression vector pBM1.1, which encodes a modified *Pseudomonas* exotoxin A gene, the fusion toxin Ki-4(scFv)-ETA´ was expressed in *E. coli* BL21 (DE3). The recombinant immunotoxin could be extracted from inclusion bodies, de- and renaturated and finally purified to a high degree (purity > 90%; Figure 6) by ion exchange chromatography.

### Specific binding and cytotoxicity of Ki-4(scFv)-ETA´

Binding activity of purified Ki-4(scFv)-ETA´ towards the target cell line L540Cy and the CD30-negative cell line BL38 as a control, was investigated by FACS analysis. Ki-4(scFv)-ETA´ binds to L540Cy cells (Figure 7B) but not to BL38 cells (Figure 7A). Specific cytotoxicity of the Ki-4(scFv)-ETA´ construct was demonstrated in a protein inhibition assay (Figure 8) for the $CD30^+$ cell line L540Cy, showing an $IC_{50}$ of 43 pM. There was no unspecific toxicity against the $CD30^-$ cell line BL38. In addition, the cytotoxicity against $CD30^+$ cells was specifically abrogated by an excess (10 μg $ml^{-1}$) of purified Ki-4 mAb but not by addition of anti-CD3 antibody OKT3 (10 μg $ml^{-1}$).

### DISCUSSION

In this paper, we report a new recombinant molecule (Ki-4 scFv) selected by phage display that binds to the CD30 antigen with high affinity. We subsequently used the Ki-4 scFv to construct a new anti-CD30 single-chain immunotoxin (Ki-4(scFv)-ETA´) by fusing the scFv to a modified *Pseudomonas* exotoxin A variant (ETA´). The major findings emerging from the present study are: (i) phage display technology allowed the identification of a high-affinity anti-CD30 Ki-4 scFv by direct selection of a hybridoma-derived phage antibody-repertoire on cells carrying the CD30 antigen; (ii) the Ki-4(scFv)-ETA´ immunotoxin shows potent and specific toxicity against the $CD30^+$ malignant Hodgkin's lymphoma cell line L540Cy ($IC_{50}$ of 43 pM).

The in vitro potency of the new single-chain immunotoxin Ki-4(scFv)-ETA´, described in the present paper, is similar to the parental IgG-based Ki-4-SMPT-dgA ($IC_{50}$ of 40 pM). Investigating the anti-CD30 antibody–antigen interaction using surface plasmon

© 1999 Cancer Research Campaign

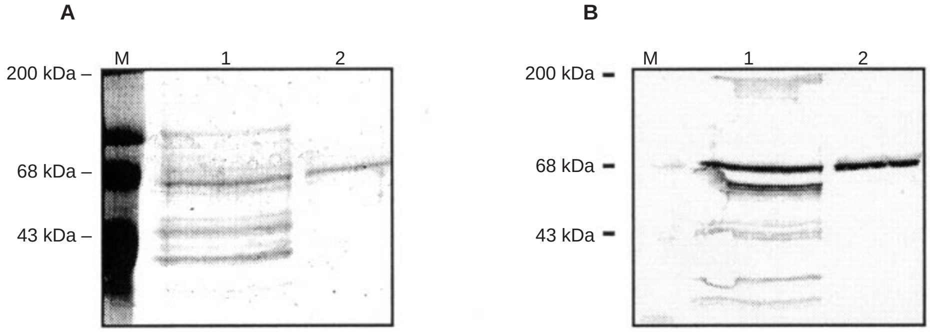

**Figure 6** Purification of Ki-4(scFv)-ETA´. The SDS-PAGE analysis was performed loading unpurified protein solution (lane 1), eluate of mono-Q-purification (lane 2) and prestained molecular weight marker (lane M). The gel was stained with Coomassie (A) and a Western blot was subsequently performed using an anti-ETA mouse IgG, anti-mouse mAb conjugated with streptavidin–alkaline phosphatase and phosphatase substrate to detect the recombinant Ki-4(scFv)-ETA´

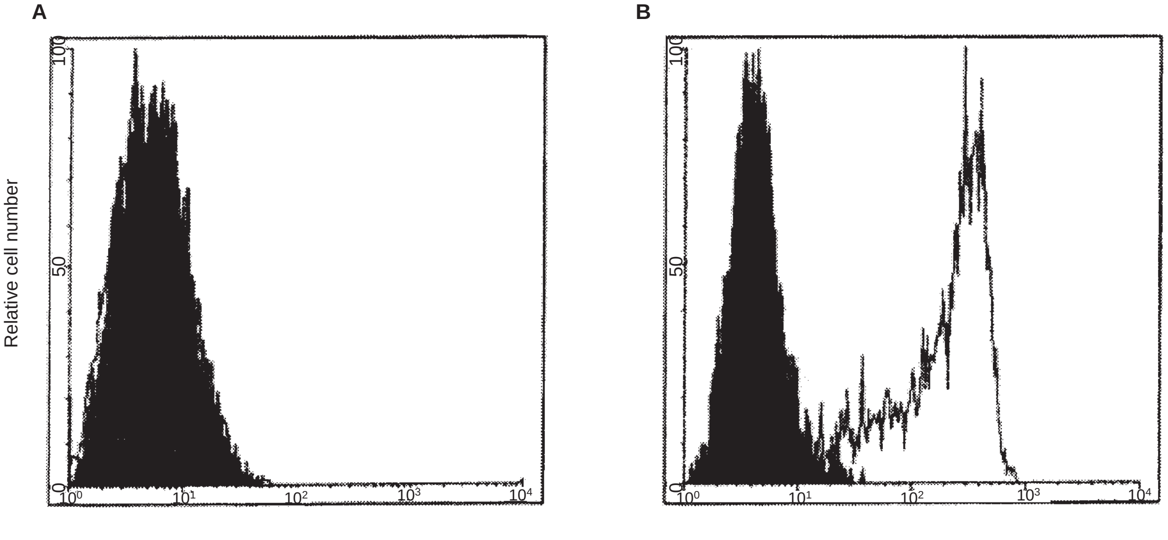

**Figure 7** Binding of Ki-4(scFv)-ETA´ to CD30-negative cell line BL38 (**A**) and CD30-positive Hodgkin-derived cell line L540Cy (**B**). Cells were stained with Ki-4(scFv)-ETA´, anti-ETA-mouse IgG (TC-1) and FITC-conjugated anti-mouse IgG (white curves) or as negative controls with PBS instead of Ki-4(scFv)-ETA´ (black curves). Histograms represent logarithms of FITC-fluorescence on flow cytometer resonance (SPR) in a BIAcore showed that Ki-4 mAb has a similar off-rate compared to Ki-4 scFv (Table 1), indicating that both immunotoxins most likely have similar binding affinities for CD30. The fact that the monovalent Ki-4(scFv)-ETA´ is highly specific and cytotoxic, indicates that cross-linking of the target antigen CD30 does not seem to be necessary for the immunotoxin to be internalized. Endocytosis of the CD30-receptor is more likely a regular process, probably in connection with a continuous recycling of the CD30 receptor. Nevertheless, the cytotoxic effect of the recombinant, monovalent immunotoxin may be further increased by creating a bivalent single-chain Fv fused to the toxin, which would possibly benefit from avid binding and enhanced internalization efficacy.

Surprisingly, no functional recombinant anti-CD30 immunotoxin has yet been published, although the construction of recombinant antibody fragments against cell surface markers and immunotoxins against a variety of malignant cells has been reported (Kreitman and Pastan, 1994). Reasons for the difficulty in obtaining functional, recombinant scFv by cloning V-genes from hybridomas are errors introduced during PCR amplification, alterations in the genes introduced by the primers used, and cloning artefacts including deletions, recombinations, insertions or frameshifts. In addition, the presence of non-functional pseudogenes or aberrantly expressed V gene transcripts of the hybridoma fusion partner can critically reduce the proportion of functional scFv (Bradbury et al, 1995). Indeed, a screening of 94 clones with about 90% containing fully scFv inserts, revealed no functional scFv. Therefore we performed two successive rounds of phage selection using a mini-repertoire of $10^5$ scFv clones to enrich a binding Ki-4 scFv clone to 95%. Rather than selecting the phage repertoire on purified CD30 antigen, we used whole cells with intact native CD30. We retrieved functional scFv fragments, which could not be detected in the starting repertoire, by simple cycles of cell panning, reamplification and phage rescue. Major advantages of this approach is that no well-characterized, purified cell surface antigen is needed and that the resulting antibody recognizes the naturally folded protein. We have recently extended this method of cell panning with phage scFv libraries, to derive a human antibody to CD30, in which the murine Ki-4 scFv was converted into a fully human version by ‘guided’ cell selection (manuscript in preparation).

 © 1999 Cancer Research Campaign

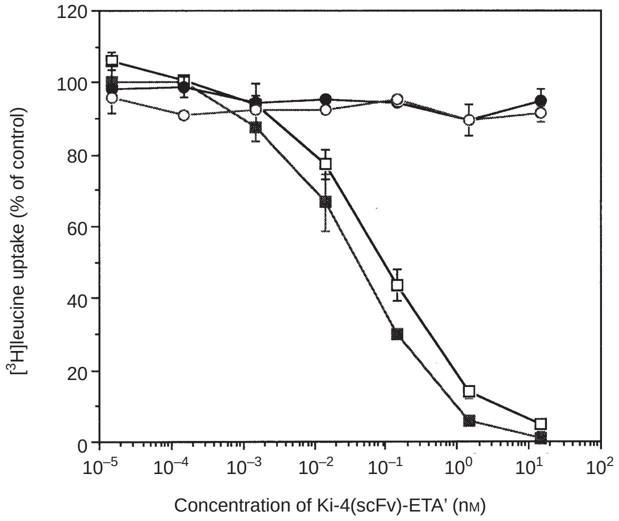

**Figure 8** Cytotoxic activity of Ki-4(scFv)-ETA´ on indicated cell lines and its competition with parental Ki-4 mAb. BL38 cells –○– and L540Cy cells –■– were incubated with a tenfold dilution series of Ki-4(scFv)-ETA´. L540Cy cells were incubated under the same conditions but with the addition of 10 µg anti-CD3 mAb OKT3 –□– or 10 µg Ki-4 mAb –●–, respectively. The incorporation of [$^3$H]leucine was measured after 48 h of treatment with various concentrations of the recombinant IT. The results are expressed as the percentage of untreated control cells

There have been very few reports on the use of phage display to isolate single-chain variable domains for the construction of recombinant immunotoxins. Lorimer et al produced a *Pseudomonas* exotoxin-based immunotoxin by fusing a scFv specific for EGFRvIII selected from a phage display library (Lorimer et al, 1996). Their construct also showed specific toxicity against transfected malignant glioblastoma cells ($IC_{50}$ of 110–160 pM). Our new Ki-4(scFv)-ETA´ immunotoxin matches the anti-EGFRvIII construct, in terms of specific toxicity ($IC_{50}$ of 43 pM). Thus, the phage display technology seems to make the isolation of specific binding moieties for the construction of highly potent recombinant immunotoxins feasible.

In summary, the newly constructed recombinant anti-CD30 immunotoxin Ki-4(scFv)-ETA´ is highly effective and specific against $CD30^+$ H-RS cells and is therefore a good candidate, either on its own or in combination with other immunotoxins for clinical evaluation in patients with $CD30^+$ lymphoma.

### ACKNOWLEDGEMENTS

The authors thank Mrs G Schön and Mrs S Drillich from the University Hospital of Cologne for their excellent technical assistance. We thank Dr J Lutgerink and Dr F Bot from the University Hospital of Maastricht for valuable discussions on whole cell ELISA and Ki-4 immunohistochemistry respectively. This work was supported in part by the 'Deutsche Forschungsgemeinschaft SFB 502' and by 'Boehringer Ingelheim Fond'.

### REFERENCES

Adams GP, McCartney JE, Tai MS, Oppermann H, Huston JS, Stafford WF, Bookman MA, Fand I, Houston LL and Weiner LM (1993) Highly specific in vivo tumor targeting by monovalent and divalent forms of 741F8 anti-c-erbB-2 single-chain Fv. *Cancer Res* **53**: 4026–4034

Barth S, Huhn M, Wels W, Diehl V and Engert A (1998) Construction and in vitro evaluation of RFT5(scFv)-ETA´, a new recombinant single-chain immunotoxin with specific cytotoxicity toward CD25+ Hodgkin-derived cell lines. *Int J Mol Med* **1**: 249–256

Bradbury A, Ruberti F, Werge T, Amati V, Di Luzio A, Gonfloni S, Hoogenboom HR, Piccioli P, Biocca S and Cattaneo A (1995) The cloning of hybridoma V regions for their ectopic expression in intracellular and intercellular immunization. In *Antibody Engineering*, 2nd edn, Borrebaeck C (ed), p. 295. Oxford University Press: New York

Dower WJ, Miller JF and Ragsdale CW (1988) High efficiency transformation of *E. coli* by high voltage electroporation. *Nucleic Acids Res* **16**: 6127–6145

Dürkop H, Latza U, Hummel M, Eitelbach F, Seed B and Stein H (1992) Molecular cloning and expression of a new member of the nerve growth factor receptor family that is characteristic for Hodgkin's disease. *Cell* **68**: 421–427

Engert A, Burrows F, Jung W, Tazzari PL, Stein H, Pfreundschuh M, Diehl V and Thorpe P (1990) Evaluation of ricin A-chain containing immunotoxins directed against CD30 as potential reagents for the treatment of Hodgkin's disease. *Cancer Res* **50**: 84–88

Engert A, Gottstein C, Bohlen H, Winkler U, Schön G, Manske O, Schnell R, Diehl V and Thorpe P (1995) Cocktails of ricin A-chain immunotoxins against different antigens on Hodgkin and Sternberg-Reed cells have superior antitumor effects against H–RS cells in vitro and solid Hodgkin tumors in mice. *Int J Cancer* **63**: 304–309

Engert A, Diehl V, Schnell R, Radzuhn A, Hatwig MT, Drillich S, Schön G, Bohlen H, Tesch H, Hansmann ML, Barth S, Schindler J, Ghetie V, Uhr J and Vitetta ES (1997) A phase-I study of an anti-CD25 ricin A-chain immunotoxin (RFT5-SMPT-dgA) in patients with refractory Hodgkin's lymphoma. *Blood* **89**: 403–410

Ghetie MA, Tucker K, Richardson J, Uhr JW and Vitetta ES (1992) The anti-tumor activity of an anti-CD22 immunotoxin in SCID mice with disseminated Daudi lymphoma is enhanced by either an anti-CD19 antibody or an anti-CD19 immunotoxin. *Blood* **80**: 2315–2320

Grossbard ML, Gribben JG, Freedman AS, Lambert JM, Kinsella J, Rabinowe SN, Eliseo L, Taylor JA, Blättler WA, Epstein CL and Nadler LM (1993) Adjuvant immunotoxin therapy with anti-B4-blocked ricin after autologous bone-marrow transplantation for patients with B-cell non Hodgkin's lymphoma. *Blood* **81**: 2263–2271

Gruss HJ and Dower SK (1995) The TNF ligand superfamily and its relevance for human disease. *Cytokines Molec Ther* **1**: 75–105

Hoogenboom HR, Griffiths AD, Johnson KS, Chiswell DJ, Hudson P and Winter G (1991) Multi-subunit proteins on the surface of filamentous phage: methodologies for displaying antibody (Fab) heavy and light chains. *Nucleic Acids Res* **19**: 4133–4137

Horn-Lohrens O, Tiemann M, Lange H, Kobarg J, Hafner M, Hansen H, Sterry W, Parwaresch RM and Lemke H (1995) Shedding of the soluble form of CD30 from the Hodgkin-analogous cell line L540 is strongly inhibited by a new CD30-specific antibody (Ki-4). *Int J Cancer* **60**: 539–544

Klimka A, Barth S, Drillich S, Wels W, van Snick J, Renauld JC, Tesch H, Bohlen H, Diehl V and Engert A (1996) A deletion mutant of *Pseudomonas* exotoxin-A fused to recombinant human interleukin-9 (rhIL-9-ETA´) shows specific cytotoxicity against IL-9-receptor-expressing cell lines. *Cytokines Mol Ther* **2**: 139–146

Kreitman RJ and Pastan I (1994) Recombinant toxins. *Adv Pharmacol* **28**: 193–219

Lorimer IAJ, Keppler-Hafkemeyer A, Beers RA, Pegram CN, Bigner DD and Pastan I (1996) Recombinant immunotoxins specific for a mutant epidermal growth factor receptor: targeted with a single chain antibody variable domain isolated by phage display. *Proc Natl Acad Sci USA* **93**: 14815–14820

Marks JD, Hoogenboom HR, Bonnert TP, McCafferty J, Griffiths AD and Winter G (1991) Bypassing immunization: human antibodies from V-gene libraries displayed on phage. *J Mol Biol* **222**: 581–597

Matthey B, Engert A, Klimka A, Diehl V and Barth S (1999) A new series of pET-derived vectors for high efficiency expression of Pseudomonas Exotoxin-based fusion proteins. *Genes* (submitted)

McCafferty J, Fitzgerald KJ, Earnshaw J, Chiswell DJ, Link J, Smith R and Kenten J (1994) Selection and rapid purification of murine antibody fragments that bind a transition-state analog by phage display. *Appl Biochem Biotechnol* **47**: 157–171

Munro S and Pelham HR (1986) An Hsp70-like protein in the ER: identity with the 78 kD glucose-regulated protein and immunoglobulin heavy chain binding protein. *Cell* **46**: 291–300

Pasqualucci L, Flenghi L, Terenzi A, Bolognesi A, Stirpe F, Bigerna B and Falini B (1995) Immunotoxin therapy of hematological malignancies. *Haematologica* **80**: 546–556

Plaksin D, Chacko S, McPhie P, Bax A, Padlan EA and Margulies DH (1996) A T cell receptor Vα domain expressed in bacteria: does it dimerize in solution? *J Exp Med* **184**: 1251–1258

Roovers RC, Henderikx P, Helfrich W, van der Linden E, Reurs A, de Bruine A, Arends J-W, de Leij L and Hoogenboom HR (1998) High affinity recombinant phage antibodies to the pan-carcinoma marker epithelial glycoprotein-2 for tumour targeting. *Br J Cancer* **78**: 1407–1416

Sahin U, Hartmann F, Senter P, Pohl C, Engert A, Diehl V and Pfreundschuh M (1990) Specific activation of the prodrug mitomycin phosphate by a bispecific anti-CD30/anti-alkaline phosphatase monoclonal antibody. *Cancer Res* **50**: 6944–6948

Schnell R, Linnartz C, Katouzi AA, Schön G, Bohlen H, Horn-Lohrens O, Parwaresch RM, Lange H, Diehl V, Lemke H and Engert (1995) A development of new ricin A-chain immunotoxins with potent anti-tumor effects against human Hodgkin cells in vitro and disseminated Hodgkin tumors in SCID mice using high-affinity monoclonal antibodies directed against the CD30 antigen. *Int J Cancer* **63**: 238–244

Schwab U, Stein H, Gerdes J, Lemke H, Kirchner H, Schaadt M and Diehl V (1982) Production of a monoclonal antibody specific for Hodgkin and Sternberg-Reed cells of Hodgkin's disease and a subset of normal lymphoid cells. *Nature* **299**: 65–67

Terenzi A, Bolognesi A, Pasqualucci L, Flenghi L, Pileri S, Stein H, Kadin M, Bigerna B, Polito L, Tazzari PL, Martelli MF, Stirpe F and Falini B (1996) Anti-CD30 (BerH2) immunotoxins containing the type-1 ribosome-inactivating proteins momordin and PAP-S (pokeweed antiviral protein from seeds) display powerful anti-tumor activity against CD30+ tumour cells in vitro and in SCID mice. *Brit J Haematol* **92**: 872–879

Vitetta E, Thorpe PE and Uhr JW (1993) Immunotoxins: magic bullets or misguided missiles? *Immunol Today* **14**: 252–259

Wels W, Harwerth IM, Müller M, Groner B and Hynes NE (1992) Selective inhibition of tumor cell growth by a recombinant single-chain antibody-toxin specific for the *erb*B-2 receptor. Cancer Res **52**: 6310–6317

Wiley SR, Goodwin RG and Smith CA (1996) Reverse signaling via CD30 ligand. *J Immunol* **157**: 3635–3639

 *© 1999 Cancer Research Campaign*
